# Relationship between right and left ventricle function in subjects free of cardiovascular diseases: a population-based MRI study

**DOI:** 10.1038/s41598-025-30588-z

**Published:** 2026-01-29

**Authors:** Ricarda von Krüchten, Roberto Lorbeer, Susanne Rospleszcz, Annette Peters, Stefan Karrasch, Holger Schulz, Bernard E. Bulwer, Charlotte Wintergerst, Esther Askani, Thierno D. Diallo, Fabian Bamberg, Christopher L. Schlett, Blerim Mujaj

**Affiliations:** 1 Department of Diagnostic and Interventional Radiology, Medical Center, Faculty of Medicine, University of Freiburg, Hugstetter Straße 55, 79106 Freiburg, Germany; 2 Department of Radiology, University Hospital of Munich, Ludwig-Maximilians-University, Marchioninistraße 15, 81377 Munich, Germany; 3 Chair of Epidemiology, Institute for Medical Information Processing, Biometry and Epidemiology, Medical Faculty, Ludwig-Maximilians-University Munich, Marchioninistr. 15, 81377 Munich, Germany; 4 Institute of Epidemiology, Helmholtz Zentrum München - German Research Center for Environmental Health, Ingolstädter Landstraße 1, 85764 Neuherberg, Germany; 5 German Center for Diabetes Research (DZD), Partner Site Neuherberg, Ingolstädter Landstraße 1, 85764 Neuherberg, Germany; 6 Institute and Outpatient Clinic for Occupational, Social and Environmental Medicine, University Hospital of Munich, Ludwig-Maximilians-University, Ziemssenstraße 1, 80336 Munich, Germany; 7 Member of the German Center for Lung Research, Comprehensive Pneumology Center Munich (CPC-M), Max-Lebsche-Platz 31, 81377 München, Germany; 8 Noninvasive Cardiology - Echocardiography Department, Cardiovascular Division, Brigham and Women’s Hospital, Boston, MA USA; 9 General Practice, Huisartsenpraktijk, Bremtstraat 116, 9320 Aalst, Belgium

**Keywords:** Cardiovascular imaging, Cardiac function, Lung volumes, Magnetic resonance imaging, Epidemiology, Cardiovascular biology, Epidemiology, Cardiology

## Abstract

Right (RV) and left ventricular (LV) volumetric measurements by cardiac magnetic resonance imaging (MRI) are established for assessing systolic and diastolic function, but the role of MRI-derived lung volumes in LV function remains unclear. This study investigated the relationship between RV and LV function, considering lung volumes. In the KORA-MRI cohort, 361 subjects underwent 3 T whole-body MRI. Cardiac functional parameters were measured from cine-steady-state free precession sequences using cvi42. Lung volumes were derived semi-automatically with an in-house algorithm. Linear regression analyses assessed RV-LV relationships, adjusted for age, sex, cardiovascular risk factors, and lung volumes. Among 361 subjects (mean age 56.1 ± 9.1 years; 43% women), RV end-diastolic volume was positively associated with LV end-diastolic (β = 28.1, p < 0.001), end-systolic (β = 11.0, p < 0.001), and stroke volume (β = 17.0, p < 0.001), but inversely with ejection fraction (β = -1.4, p = 0.001). RV end-systole was positively associated with LV end-diastolic (β = 21.2, p < 0.001), end-systolic (β = 11.5, p < 0.001), stroke volume (β = 9.7, p < 0.001), and inversely with ejection fraction (β = -3.3, p < 0.001). Adjusting for lung volumes did not alter RV-LV associations, and no effect modification by sex was observed despite lung volume differences. In individuals without cardiovascular disease, RV and LV volumetric parameters were strongly associated, supporting the critical role of RV function in LV function, independent of lung volumes.

**Supplementary Information:**

The online version contains supplementary material available at 10.1038/s41598-025-30588-z.

## Introduction

Global cardiac function assessment relies on the measurement of diastolic and systolic function of the left ventricle (LV), with LV ejection fraction (LVEF) being the most widely used^1^. The LVEF is a well-established parameter used for the diagnosis, clinical decision-making, and risk stratification of patients with cardiovascular diseases, with echocardiography^2,3^, computed tomography (CT)^4^, and magnetic resonance imaging (MRI)^5^ being the primary diagnostic tools. The LVEF represents the volume of blood ejected into the systemic circulation during each contractile cycle, with right ventricle (RV) ejection fraction serving the pulmonary circulation, which includes the lungs and pulmonary vasculature. During diastole, RV and pulmonary pressures are directly exposed to LV filling pressures, and more broadly to global LV performance. The LV can maintain the systemic circulation even in the presence of RV dysfunction^6^. However, with diseases of the pulmonary vasculature and in the presence of impaired LV systolic or diastolic function, RV function becomes essential for maintaining cardiac output^7^. Therefore, right-heart function, which involves the RV, the pulmonary circulation, and lung function, including lung volumes, impacts LV function and vice versa. This becomes clinically evident in patients with cor pulmonale, heart failure with reduced LV ejection fraction (HFrEF), and heart failure with preserved LV ejection fraction (HFpEF).

The relationships between LV and RV function, the pulmonary circulation, and lung parameters, including lung mass and volumes, as well as compensatory mechanisms involved with impaired cardiac function, are not well characterized^8^. Whether lung volumes directly impact or modify RV and LV function is unclear. Moreover, evidence of whether RV function influences LV function directly or through the pulmonary circulation remains sparse. From a community screening perspective, asymptomatic or at-risk patients with HFpEF, elevated LV filling pressures due to LV stiffness and impaired LV filling could be reflected in the pulmonary vasculature and lung function, thereby impacting lung volumes and RV function. Whole-body MRI has the advantage of simultaneous assessment of both lung volumes and volumetric LV and RV cardiac function within a single MRI scan^9^. To date, no study has simultaneously investigated these relationships.

Therefore, we aimed to investigate the relationship between lung volumes and global LV and RV function in subjects free of cardiovascular diseases within the population-based Cooperative Health Research in the Region of Augsburg (KORA)-MRI study.

## Results

Table 1 summarizes the characteristics of the study population, with a mean age of 56.1 ± 9.1 years, and 43% of the subjects were women. Diabetes mellitus was prevalent in 12.5% of subjects. Median fasting glucose was 5.5 mmol/L, and median fasting insulin was 54.7 pmol/L. Hypertension was present in 33.2% of subjects, and 24.9% of subjects were on antihypertensive medication. Among the subjects, 20.8% were current smokers, the mean BMI was 28.0 ± 4.8, and the mean BSA was 1.95 ± 0.22. In all subjects, the mean lung volume was 3.98 ± 1.12, and the prevalence of a history of COPD was 4.7%. The mean eGFR was 87.1 ± 13. According to categories of lung volume tertiles, RV end-diastole, end-systole, and ejection fraction differed significantly between low, middle, and high tertiles. For LV, subjects in the high tertile of lung volumes had higher LV mass compared to middle and low tertile (Supplementary Table S1).

**Table 1:** Study population characteristics.

	All	Female	Male	P
N	361	154 (43%)	207 (57%)	
Age, years	56.1 (± 9.1)	56.1 (± 9.1)	56.1 (± 9.1)	0.977
Body mass index, kg/m^2^	28.0 (± 4.8)	27.6 (± 5.5)	28.2 (± 4.2)	0.247
Body surface area, m^2^	1.95 (± 0.22)	1.79 (± 0.17)	2.07 (± 0.17)	< 0.001
Smoking status				0.136
Never, (%)	131 (36.3%)	63 (40.9%)	68 (32.9%)	
Past, (%)	155 (42.9%)	57 (37.0%)	98 (47.3%)	
Current, (%)	75 (20.8%)	34 (22.1%)	41 (19.8%)	
Alcohol use, (g/day)	18.3 (± 22.3)	8.8 (± 14.3)	25.3 (± 24.4)	< 0.001
History of COPD	17 (4.7%)	7 (4.6%)	10 (4.8%)	0.899
Diabetes status				0.019
Normal (%)	222 (61.5%)	107 (69.5%)	115 (55.6%)	
Prediabetes (%)	94 (26%)	34 (22.1%)	60 (29%)	
Diabetes (%)	45 (12.5%)	13 (8.4%)	32 (15.5%)	
Fasting glucose, mmol/L	5.50 (5.11;6.05)	5.27 (4.94;5.88)	5.64 (5.27;6.16)	< 0.001
Fasting insulin, pmol/L	54.7 (37.2;81.0)	49.3 (35.4;78.0)	60 (38.3;87.1)	0.010
HOMA-index	2.20 (1.41;3.51)	1.96 (1.29;3.33)	2.55 (1.52;3.66)	0.004
Hypertension, (%)	120 (33.2%)	43 (27.9%)	77 (37.2%)	0.064
Systolic blood pressure, mm/Hg	120.3 (± 16.8)	112.9 (± 14.5)	125.9 (± 16.3)	< 0.001
Diastolic blood pressure, mm/Hg	75.2 (± 9.9)	72.0 (± 8.6)	77.6 (± 10.2)	< 0.001
Antihypertensive medication, (%)	90 (24.9%)	41 (26.6%)	49 (23.7%)	0.521
Total cholesterol, mmol/L	5.61 (± 0.95)	5.63 (± 0.9)	5.59 (± 0.98)	0.646
HDL Cholesterol, mmol	1.61 (± 0.46)	1.82 (± 0.46)	1.45 (± 0.38)	< 0.001
LDL Cholesterol, mmol	3.58 (± 0.86)	3.50 (± 0.83)	3.64 (± 0.88)	0.130
Triglycerides, mmol	1.21 (0.87;1.75)	1.08 (0.77;1.37)	1.38 (0.97;2.12)	< 0.001
Lipid lowering medication, %	39 (10.8%)	18 (11.7%)	21 (10.1%)	0.640
eGFR, ml/min/1.73 m^2^	87.1 (± 13)	85.7 (± 13.3)	88.1 (± 12.7)	0.093
Lung Volumes, L	3.98 (± 1.12)	3.31 (± 0.71)	4.48 (± 1.10)	< 0.001
cardiac parameters				
RV End-diastolic Volume, (mL)	165.6 (± 39.6)	144.2 (± 30.9)	181.4 (± 38.0)	< 0.001
RV End-systolic Volume, (mL)	79.5 (± 25.8)	64.5 (± 18.9)	90.7 (± 24.6)	< 0.001
RV Stroke Volume, (mL)	86.1 (± 19.5)	79.9 (± 17.1)	90.8 (± 20.0)	< 0.001
RV Ejection fraction, (%)	52.6 (± 7.1)	55.7 (± 6.2)	50.3 (± 6.8)	< 0.001
LV End-diastolic Volume, (mL)	130.6 (± 32.5)	118.2 (± 26.1)	139.8 (± 33.8)	< 0.001
LV End-systolic Volume, (mL)	41.2 (± 18.1)	34.8 (± 14.7)	46.0 (± 19.0)	< 0.001
LV Stroke Volume, (mL)	89.4 (± 20.1)	83.4 (± 16.9)	93.9 (± 21.2)	< 0.001
LV Ejection fraction, (%)	69.2 (± 7.8)	71.1 (± 6.7)	67.8 (± 8.3)	< 0.001
LV Peak ejection rate, (mL/s)	357.8 (± 133.6)	334.1 (± 107.3)	375.5 (± 148)	0.003
LV Early diastolic filling rate, (mL/s)	230.4 (± 115.5)	231.5 (± 106.4)	229.6 (± 122.1)	0.875
LV Late diastolic filling rate, (mL/s)	240.6 (± 141)	236.3 (± 134)	243.9 (± 146.2)	0.614
LV Mass, diastolic, g	141 (± 34.6)	114.5 (± 23.9)	160.6 (± 27.5)	< 0.001

The values represent mean ± standard deviation (SD), median (interquartile ranges) or frequency along with percentage (%). P = p-value for difference (t-test, Mann–Whitney-U test or chi^2^-test); Abbreviation: eGFR = estimated glomerular filtration rate, HDL = high-density lipoprotein, HOMA-index = homeostasis model assessment –index, LDL = low-density lipoprotein; LV = left ventricle; RV = right ventricle.

### Association between RV and LV function parameters

We found significant associations between RV and LV functional parameters. RV end-diastolic volume was positively associated with LV end-diastolic volume, systolic volume, stroke volume, peak ejection rate, early diastolic rate, late diastolic rate, and mass, and inversely associated with ejection fraction (Table 2). RV end-systolic volume was positively associated with LV end-diastolic volume, systolic volume, stroke volume, peak ejection rate, early diastolic rate, and mass, and inversely associated with ejection fraction, but not with late diastolic rate (Table 2). RV stroke volume was positively associated with LV end-diastolic volume, systolic volume, stroke volume, ejection fraction, peak ejection rate, early diastolic rate, late diastolic rate, and mass (Table 2). RV ejection fraction was associated with LV stroke volume, ejection fraction, and late diastolic rate, and inversely associated with end-systolic volume, but not with end-diastolic volume, peak ejection rate, early diastolic rate, and mass (Table 2). The association between RV and LV did not attenuate after adjustment in all models, including model 2, and no effect modification was observed by lung volumes. In sensitivity analyses by excluding subjects with a history of COPD, the results were confirmatory (Supplementary Table S2).

**Table 2:** Association between right ventricle function parameters and left ventricle function parameters.

PER SD	Model 1	P	Model 2	P	Model 3	P	Model 4	P
	LV EDV							
RV EDV	28.1 (26.0; 30.1)	< 0.001	27.9 (25.8; 29.9)	< 0.001	27.2 (25.1; 29.4)	< 0.001	26.8 (24.6; 29.1)	< 0.001
RV ESV	21.2 (18.3; 24.0)	< 0.001	21.0 (18.2; 23.9)	< 0.001	19.6 (16.7; 22.5)	< 0.001	18.9 (16.0; 21.8)	< 0.001
RV SV	25.1 (23.1; 27.1)	< 0.001	25.0 (23.0; 27.0)	< 0.001	24.5 (22.4; 26.7)	< 0.001	24.1 (21.9; 26.3)	< 0.001
RV EF	0.3 (−3.0; 3.6)	0.855	0.06 (−3.2; 3.3)	0.969	−0.2 (−3.4; 2.9)	0.87	0.1 (−3.0; 3.3)	0.922
	LV ESV							
RV EDV	11.0 (9.3; 12.7)	< 0.001	10.9 (9.2; 12.7)	< 0.001	11.1 (9.3; 12.9)	< 0.001	10.8 (9.0; 12.6)	< 0.001
RV ESV	11.5 (9.8; 13.2)	< 0.001	11.4 (9.7; 13.1)	< 0.001	11.2 (9.4; 12.9)	< 0.001	10.8 (9.1; 12.6)	< 0.001
RV SV	6.3 (4.6; 8.1)	< 0.001	6.2 (4.4; 8.0)	< 0.001	6.1 (4.2; 8.0)	< 0.001	5.8 (3.9; 7.7)	< 0.001
RV EF	−5.5 (−7.3; −3.7)	< 0.001	−5.6 (−7.4; −3.8)	< 0.001	−5.6 (−7.4; −3.8)	< 0.001	−5.3 (−7.1; −3.5)	< 0.001
	LV SV							
RV EDV	17.0 (15.6; 18.4)	< 0.001	16.9 (15.5; 18.3)	< 0.001	16.1 (14.6; 17.6)	< 0.001	16.0 (14.5; 17.5)	< 0.001
RV ESV	9.7 (7.6; 11.7)	< 0.001	9.6 (7.5; 11.6)	< 0.001	8.4 (6.4; 10.4)	< 0.001	8.0 (6.0; 10.1)	< 0.001
RV SV	18.7 (18.0; 19.5)	< 0.001	18.7 (17.9; 19.4)	< 0.001	18.4 (17.5; 19.2)	< 0.001	18.2 (17.4; 19.1)	< 0.001
RV EF	5.8 (3.7; 7.8)	< 0.001	5.6 (3.6; 7.7)	< 0.001	5.3 (3.3; 7.2)	< 0.001	5.4 (3.5; 7.3)	< 0.001
	LV EF							
RV EDV	−1.4 (−2.3; −0.5)	0.001	−1.4 (−2.3; −0.5)	0.002	−1.6 (−2.6; −0.7)	0.001	−1.5 (−2.5; −0.5)	0.002
RV ESV	−3.3 (−4.1; −2.4)	< 0.001	−3.3 (−4.1; −2.4)	< 0.001	−3.4 (−4.3; −2.5)	< 0.001	−3.3 (−4.2; −2.4)	< 0.001
RV SV	1.1 (0.2; 1.9)	0.01	1.1 (0.2; 1.9)	0.009	1.1 (0.2; 2.0)	0.011	1.2 (0.3; 2.1)	0.006
RV EF	4.0 (3.2; 4.7)	< 0.001	4.0 (3.3; 4.8)	< 0.001	3.9 (3.2; 4.7)	< 0.001	3.8 (3.1; 4.6)	< 0.001
	LV PEAK EJECTION RATE							
RV EDV	102 (90; 114)	< 0.001	102 (91; 114)	< 0.001	101 (89; 113)	< 0.001	101 (89; 113)	< 0.001
RV ESV	71.7 (57.6; 85.8)	< 0.001	71.5 (57.4; 85.6)	< 0.001	66.1 (52.0; 80.2)	< 0.001	65.2 (50.9; 79.6)	< 0.001
RV SV	98.0 (87.6; 108)	< 0.001	98.6 (88.1; 109)	< 0.001	99.7 (89.0; 110)	< 0.001	98.7 (87.7; 109)	< 0.001
RV EF	14.3 (−0.3; 28.9)	0.055	14.0 (−0.6; 28.6)	0.061	13.5 (−0.5; 27.7)	0.06	13.6 (−0.6; 27.9)	0.061
	LV EARLY DIASTOLIC RATE							
RV EDV	84.3 (74.3; 94.2)	< 0.001	83.7 (73.8; 93.7)	< 0.001	83.4 (73.3; 93.4)	< 0.001	84.0 (73.7; 94.4)	< 0.001
RV ESV	58.8 (46.9; 70.7)	< 0.001	58.3 (46.4; 70.2)	< 0.001	53.5 (41.7; 65.3)	< 0.001	53.3 (41.2; 65.4)	< 0.001
RV SV	80.8 (71.9; 89.6)	< 0.001	80.4 (71.5; 89.3)	< 0.001	82.7 (73.9; 91.6)	< 0.001	82.5 (73.4; 91.6)	< 0.001
RV EF	11.4 (−0.8; 23.7)	0.068	10.7 (−1.5; 22.9)	0.086	11.8 (0.1; 23.5)	0.048	11.7 (−0.1; 23.6)	0.054
	LV LATE DIASTOLIC RATE							
RV EDV	37.8 (21.5; 54.0)	< 0.001	36.5 (20.2; 52.7)	< 0.001	34.3 (17.0; 51.6)	< 0.001	29.3 (11.9; 46.7)	0.001
RV ESV	9.4 (−7.4; 26.2)	0.271	8.7 (−8.0; 25.4)	0.309	3.6 (−13.6; 20.9)	0.68	0.1 (−17.1; 17.4)	0.986
RV SV	54.8 (40.5; 69.1)	< 0.001	53.6 (39.2; 68.0)	< 0.001	55.9 (40.6; 71.3)	< 0.001	50.8 (35.3; 66.3)	< 0.001
RV EF	33.2 (18.1; 48.4)	< 0.001	32.4 (17.3; 47.5)	< 0.001	34.0 (18.8; 49.2)	< 0.001	32.3 (17.2; 47.5)	< 0.001
	LV MASS							
RV EDV	8.8 (5.8; 11.8)	< 0.001	8.9 (5.9; 11.9)	< 0.001	9.0 (6.2; 11.7)	< 0.001	8.5 (5.7; 11.3)	< 0.001
RV ESV	5.3 (2.2; 8.4)	0.001	5.4 (2.2; 8.5)	0.001	5.7 (2.9; 8.5)	< 0.001	5.0 (2.1; 7.8)	0.001
RV SV	9.3 (6.6; 12.0)	< 0.001	9.5 (6.7; 12.2)	< 0.001	9.0 (6.4; 11.5)	< 0.001	8.9 (6.3; 11.4)	< 0.001
RV EF	2.2 (−0.6; 5.1)	0.131	2.2 (−0.6; 5.1)	0.125	1.1 (−1.4; 3.7)	0.392	1.7 (−0.8; 4.3)	0.189

The beta estimate given with a 95% confidence interval represents the estimate size between RV and LV function from linear regression model. The model 1 = adjusted for sex and age; model 2 = model 1 + lung volumes; model 3 = model 2 + smoking, alcohol use, BMI, systolic blood pressure, diastolic blood pressure, diabetes mellitus, total cholesterol and eGFR; model 4 = model 3 + insulin, glucose, antihypertensive medication, lipid lowering medication; CI = 95% confidence interval; SD = standard deviation. Abbreviation: BMI = body mass index; EDV = end-diastolic volume; EF = ejection fraction; eGFR = estimated glomerular filtration rate, ESV = end-systolic volume; LV = left ventricle, RV = right ventricle; SV = stroke volume.

### Stratified analysis according to categories of lung volumes

**Fig. 1:**
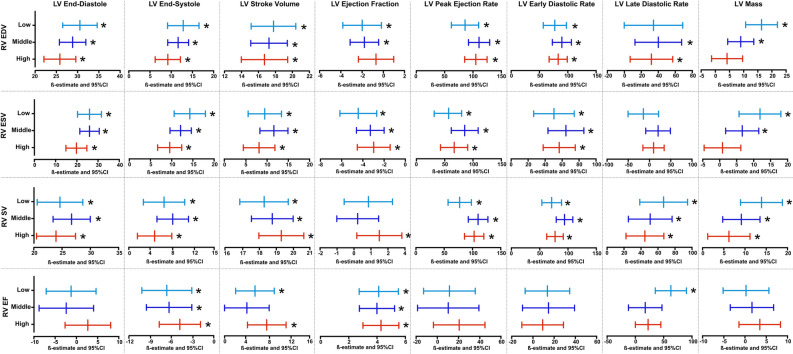
Flowchart of the study. Abbreviations: BMI = body mass index; BP = blood pressure; BSA = body surface area; CAD = cardiovascular disease; COPD = chronic obstructive pulmonary disease; DBP = diastolic blood pressure; DM = diabetes mellitus; eGFR = estimated glomerular filtration rate; HOMA-IR = homeostasis model assessment-insulin resistance index; IGT = impaired glucose tolerance; LV = left ventricle; OGTT = oral glucose tolerance test; PAD = peripheral artery disease; RV = right ventricle; SBP = systolic blood pressure.

In stratified analysis, according to lung volume tertiles (Fig. 1, Supplementary Tables S3 – S14), in subjects with higher lung volumes RV end-diastolic volume was positively associated with LV end-diastolic volume, end-systolic volume, stroke volume, peak ejection rate, early diastolic rate, but not with ejection fraction, late diastolic rate and ventricle mass. Similarly, RV end-systolic volume was associated with LV end-diastolic volume, end-systolic volume, stroke volume, peak ejection rate, early diastolic rate, and ventricle mass, inversely with ejection fraction, but not with late diastolic rate, similarly to middle and high tertiles. RV stroke volume was associated with LV end-diastolic volume, end-systolic volume, stroke volume, peak ejection rate, early diastolic rate, late diastolic rate, and ventricle mass, but not with ejection fraction, similarly to middle and high tertiles. RV ejection fraction was associated with LV stroke volume, ejection fraction, late diastolic rate, and inversely with end-systolic volume but not with end-diastolic volume, peak ejection rate, early diastolic rate, and mass, while in the middle tertile and high tertile, the association with late diastolic rate became non-significant.

### Stratified analysis according to sex

**Fig. 2:**
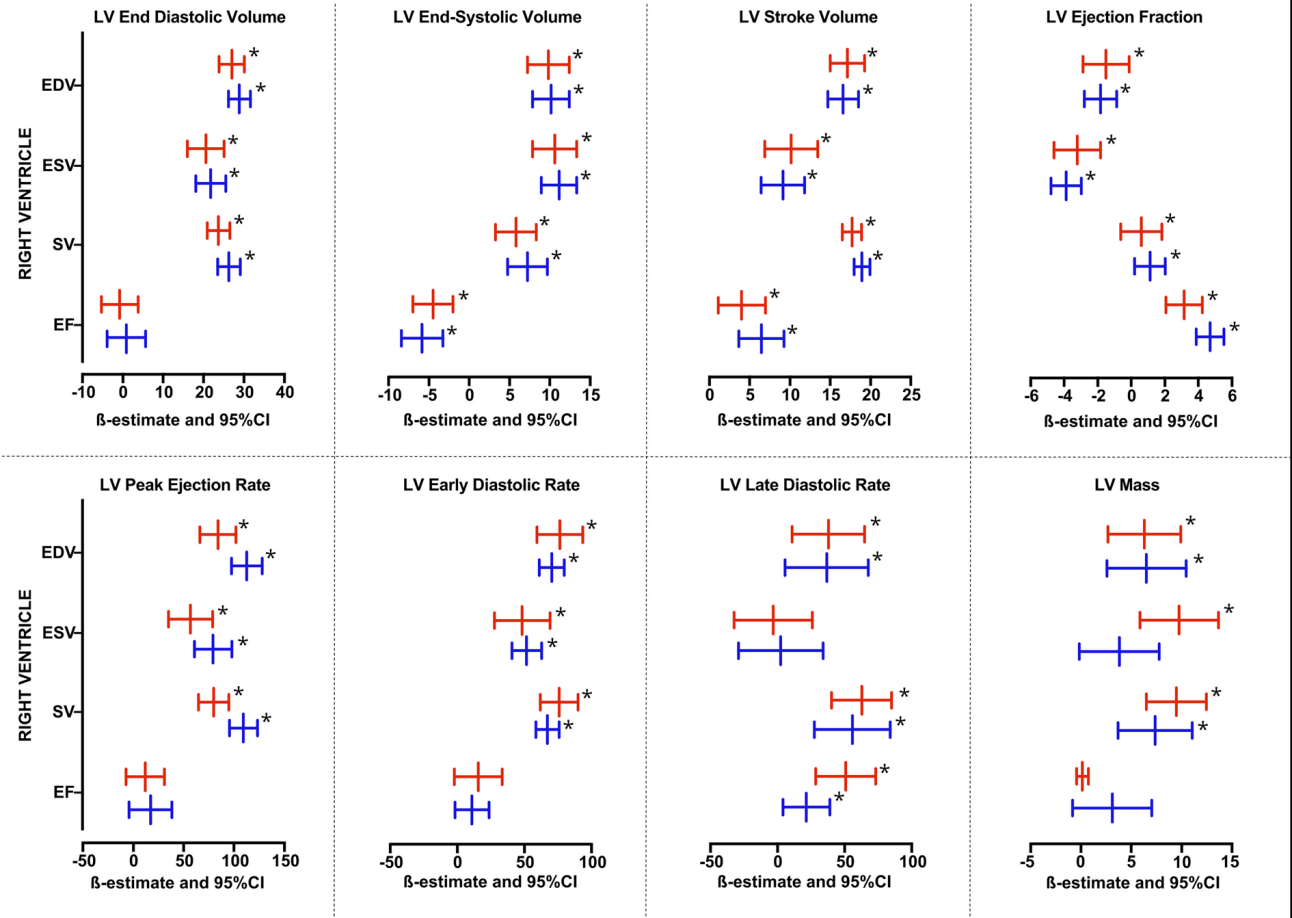
The relationship between right ventricle function parameters and left ventricle function parameters, according to tertiles of lung volumes (light blue–low, blue–middle, and red–high). The ß-estimate with 95% confidence interval depicts the effect size for each parameter from linear regression model adjusted for age and sex. Asterisks denotes p-value < 0.05.

In females (Fig. 2, Supplementary Tables S15 – S18), RV end-diastolic volume was associated with LV end-diastolic volume, end-systolic volume, stroke volume, peak ejection rate, early diastolic rate, late diastolic rate, and ventricle mass, but not with ejection fraction. RV end-systolic volume was associated with LV end-diastolic volume, end-systolic volume, stroke volume, peak ejection rate, early diastolic rate, and ventricle mass, inversely with ejection fraction, but not with late diastolic rate. RV stroke volume was associated with LV end-diastolic volume, end-systolic volume, stroke volume, peak ejection rate, early diastolic rate, late diastolic rate, and ventricle mass, but not with ejection fraction. RV ejection fraction was associated with LV stroke volume, ejection fraction, late diastolic rate, and inversely with end-systolic volume but not with end-diastolic volume, peak ejection rate, early diastolic rate, and mass. In males (Fig. 2, Supplementary Tables S19 – S22), RV end-diastolic volume was associated with LV end-diastolic volume, end-systolic volume, stroke volume, peak ejection rate, early diastolic rate, late diastolic rate, ventricle mass, and inversely with ejection fraction. RV end-systolic volume was associated with LV end-diastolic volume, end-systolic volume, stroke volume, peak ejection rate, early diastolic rate, and ventricle mass, inversely with ejection fraction, but not with late diastolic rate. RV stroke volume was associated with LV end-diastolic volume, end-systolic volume, stroke volume, ejection fraction, peak ejection rate, early diastolic rate, late diastolic rate, and ventricle mass. RV ejection fraction was associated with LV stroke volume, ejection fraction, late diastolic rate, and inversely with end-systolic volume but not with end-diastolic volume, peak ejection rate, early diastolic rate, and mass.

## Methods

### Study population

**Fig. 3:**
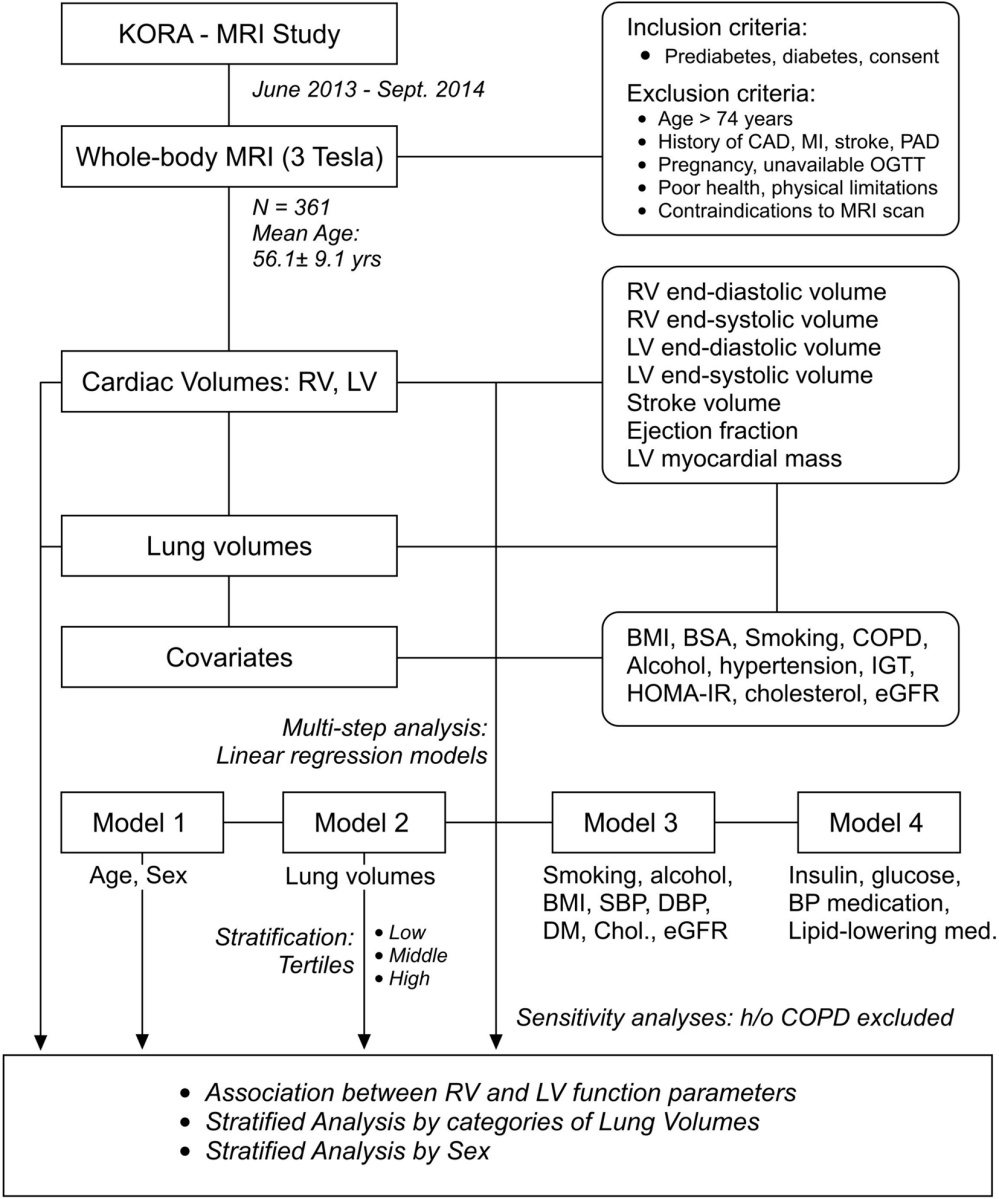
The relationship between right ventricle function parameters and left ventricle function parameters, according to sex (red–female, blue–male). The univariate ß-estimate with 95% confidence interval depicts the effect size for each parameter from linear regression model adjusted for age. Asterisks denotes p-value < 0.05.

Subjects from the region of Augsburg, Germany, aged between 25 and 74 years, were recruited in the KORA-MRI study^10^. Participants were examined at the KORA study center between June 2013 and September 2014, and a 3 Tesla whole-body MRI scan was performed^10^. Inclusion criteria for undergoing a whole-body MRI scan included prediabetes, diabetes for the control group, and written informed consent for all participants. Exclusion criteria were age > 74 years, participants with a known history of coronary artery disease, myocardial infarction, stroke, peripheral artery disease, pregnancy, unavailable oral glucose test, poor overall health condition, or other physical limitations. In addition, subjects with contraindications to MRI scan, such as known gadolinium contrast allergy, cardiac stents, cardiac pacemaker or implantable defibrillator, implanted metal parts, breast-feeding women, subjects with claustrophobia, and subjects with impaired renal function were excluded. From a total of participants with an MRI scan (n = 400), subjects with incomplete MRI data and/or inadequate image quality were excluded. Hence, 361 subjects were included in the analysis (Fig. 3).

The KORA-MRI study was compiled according to the Helsinki Declaration on Human Research^11^ and approved by the Institutional Research Ethics Board of the Medical Faculty of Ludwig-Maximilian University, Munich.

### Whole-body MR imaging protocol

A 3-Tesla MRI system (MagnetomSkyra, Siemens AG, Healthcare Sector, Erlangen, Germany)^10^ was applied to perform the whole-body MRI scans using an 18-channel body surface coil and a table-mounted spine matrix coil. The protocol included sequences covering the entire body from the head to the femur, including particular organs, e.g., brain, carotid arteries, tissue/organ quantification, and fat compartments. A 2-point DIXON T1 sequence was used to analyze the lung in submaximal inspiration breath-hold and an acquisition time of 15 s. Slice thickness was 3 mm, coronal acquisition, including a field of view (FOV) of 488 mm x 716 mm, a matrix of 256 × 256, a repetition time (TR) of 4.06 ms, and an echo time (TE) of 1.26 ms. For analysis of the heart, the cine-steady-state free precession sequence was acquired in a short-axis view with 10 layers and 25 phases. Slice thickness was 8 mm, including a FOV of 297 mm × 360 mm, a matrix of 240 × 160, a TR of 29.97 ms, a TE of 1.46 ms, and a flip angle of 62°.

### MR-Image analysis for cardiac measurements

Cine steady-state free precession (cine-SSFP) sequences were used to imaging cardiac function, morphology, and morphology diseases. LV and RV function were evaluated using commercially available Cvi42 software (version 4.1.5(190)); Circle Cardiovascular Imaging Inc. (Calgary, Alberta, Canada). Detection of LV contours and calculation of LV volumes was processed automatically, and if necessary, corrected manually according to guidelines^12^, and LV myocardial mass was assessed during end-diastole. After manually segmenting the lumen of the RV in the end-systole and end-diastole in each layer from the cardiac apex to the pulmonary valve^13^, the software automatically calculated the corresponding volumes. The difference between the end-systolic and end-diastolic volumes comprises the stroke volume and ejection fraction parameters. Furthermore, filling and ejection rates for LV were quantified using pyHeart, a dedicated in-house software displaying LV time-volume-curves^14,15^. Peak gradients were assessed during systolic ejection and early LV filling, an active process involving LV diastolic suction and recoil, and late LV filling caused by atrial contraction^16^**.**

### MR Image analysis for lung volume

An algorithm was used to automatically process the MR images for quantification of lung volumes, as described previously^9,17^. The algorithm was trained to perform the following segmentation steps: Correction of intensity inhomogeneities, pre-extraction of a coarse region of interest containing the airways, segmentation of bilateral lungs and trachea region, extraction of the trachea, separating the lung into right and left lung, and refining the lung region. Pulmonary blood vessels outside the mediastinal contours were included in the lung region^9^. An independent reader visually checked the MRI scans set after the automated processing, unaware of the clinical information, and high-quality outputs of the algorithm framework have been verified^9^.

### Covariates

Information on risk factors was obtained through physical examination, interview, and blood sampling. Body mass index (BMI) and body surface area (BSA) were calculated based on height and weight, smoking status, history of chronic obstructive pulmonary disease (COPD), alcohol use (g/day), and antihypertensive medication were assessed by questionnaire. According to the WHO criteria, diabetes state was defined as prediabetes (impaired glucose tolerance, IGT: normal fasting glucose concentration and a 2-h serum oral glucose tolerance test (OGTT) glucose concentration between 140 and 200 mg/dL; and/or an impaired fasting glucose concentration, as defined by fasting glucose levels between 110 and 125 mg/dL, and a normal 2-h serum glucose concentration), and diabetes (2-h serum glucose concentration as determined by OGTT that was > 200 mg/dL and/or a fasting glucose level that was > 125 mg/dL)^18^.

The homeostasis model assessment-insulin resistance index (HOMA-IR) was calculated using the formula: [fasting glucose (mmol/L) x fasting insulin (µU/L)/22.5]^19^. Hypertension was defined as systolic blood pressure > 140 mmHg, diastolic blood pressure > 90 mmHg, or receiving current antihypertensive treatment. Using an enzymatic colorimetric method (Dimension Vista 1500, Siemens Healthcare Diagnostics, Eschborn, Germany, or Cobas c702, Roche Diagnostics GmbH, Mannheim, Germany) the total serum cholesterol and serum creatinine concentrations were analyzed. The estimated glomerular filtration rate (eGFR) was calculated based on creatinine/cystatin C or combination according to a standardized formula^20^.

### Statistical analysis

The distribution of population characteristics was described by using mean and standard deviation (SD), median (interquartile ranges (IQRs), or percentages for continuous and categorical variables, respectively. We used a multi-step approach analysis, first using linear regression models, we investigated the association between RV and LV functional parameters. In Model 1, we adjusted for sex and age. Model 2 was adjusted for lung volumes, and Model 3 additionally for smoking, alcohol use, BMI, systolic blood pressure, diastolic blood pressure, diabetes mellitus, total cholesterol, and eGFR. Model 4 was additionally adjusted for insulin, glucose, antihypertensive, lipid-lowering medication. Second, by stratifying the subjects per lung volumes (tertiles) categories in low, middle, and high, using the above-mentioned linear regression models, we assessed the association between RV and LV function parameters. Third, stratified analyses for sex were performed using the same models in linear regression analysis. In sensitivity analyses, subjects with a history of COPD were excluded, and the same models in linear regression analysis were performed. A p-value of < 0.05 was considered statistically significant. All analyses were performed using Stata (Stata 16.1 Corporation, College Station, TX, USA).

## Discussion

In subjects free of cardiovascular diseases, RV function was associated with LV function independently of lung volumes. Furthermore, lung volumes did not modify the relationship between RV and LV. Despite dissimilar hemodynamics, especially the marked differential between right- and left-sided intracardiac pressures, these findings confirm strong coupling between the RV and LV. Therefore, although the lungs are a constituent part of pulmonary circulation, LV function parameters remain closely coupled with the RV’s.

Previously, our group investigated the relationship between RV and LV with lung volumes, with evidence that LV stroke volume and LV early diastolic filling rate were inversely associated with lung volumes^15^. This suggests that lung volumes may be an effect modifier or confounder of LV function. Given the previous observations on the possible association between lung volumes with RV and LV^15^, we tested the hypothesis of effect modification.

Normal LV diastolic filling is an active process (LV diastolic suction) involving active diastolic recoil and relaxation at normal filling pressures. Although this is intimately reflected in the pulmonary circulation and RV function, the degree to which pulmonary function, specifically lung volumes, impacts RV and LV remains unclear. To date, our study is the first study to simultaneously assess the relationship of cardiac volumes along with lungs as volumetric tissue mass. In the previous population-based MESA study, the effect of pulmonary vasculature on pulmonary function and dyspnea was investigated. This study reported that lower pulmonary vascular volume was associated with lower LV end-diastolic volume, stroke volume, and cardiac output among subjects with a long-term smoking history^21^. Similarly, within the MESA study, the role of a greater extent of emphysema was assessed, and this study reported that airflow obstruction was linearly related to impaired LV filling and overall lower cardiac output^22^. The study sheds light on structural respiratory changes in advanced respiratory diseases where the lungs can directly trigger compensatory mechanisms to increase the lung mass and compensate for oxygenation capacity. However, there was no evidence on RV functional capacity from emphysema patients undergoing surgery, although a recent study showed that the effect of lung volume reduction improved LV filling and cardiac performance^23^. In this context, our findings provide additional evidence combining information from three key organs: the RV, the lung, and the LV.

Whole-body MRI, and its capacity to simultaneously assess both pulmonary and global cardiac function, could potentially deliver distinct advantages for assessing patients at risk for developing^24^. Cardiometabolic risk factors (e.g., hypertension, diabetes, dyslipidemia, and smoking) and increasing age are major risk factors for HFpEF, and such patients often pose a diagnostic and therapeutic challenge as they present with pulmonary symptoms (dyspnea and exertional fatigue), but with normal LV volumes and ejection fraction. Hence, the simultaneous assessment of both pulmonary and cardiac parameters using whole-body MRI is potentially promising in this population.

Given the fact that the vast majority of participants were free of active respiratory disease (95.3%), lung volume differences were observed, and all participants had preserved cardiac volumes. The relationship between RV and LV was examined by adjusting lung volumes in an attempt to explore the modification mechanisms of lung volumes on the relationship between RV and LV. Observations revealed that, despite the potential modification by lung volumes, RV and LV function parameters remain coupled. However, our results should not be interpreted in the context of pulmonary blood flow or pulmonary microvascular measurements^21^.

The complex relationship between cardiac volumetric parameters and lung volumes may be explained by various risk factors affecting cardiac function, such as smoking^21,25^ and environmental factors^26^, or by the influence of risk factors such as hypertension^27^ and diabetes mellitus^28^. Previously, we investigated the role of serum insulin on lung volume and observed that increased serum insulin levels were associated with decreased lung volume and RV function^16^. This confirms that the negative influence of various factors affects heart and lung function independently, and compensatory changes may not affect their mutual function^29^.

The strengths of the study include the use of advanced 3 T whole-body MRI technology with a detailed protocol that included a cine-steady-state free precession sequence for imaging of the heart and lungs among healthy individuals free of cardiovascular disease from a population-based cohort. In addition, whole-body MRI allows simultaneous assessment of the heart and lungs within a single time point for reliable real-time cardio-pulmonary function evaluation. Further, automatic algorithms were used for MR image analysis of lung volumes and heart function volumes quantification. Furthermore, multivariable adjustment, stratified analysis, and sensitivity analysis by excluding COPD patients were applied to confirm the results. However, our study encounters limitations that need to be addressed. First, although the MRI protocol for cardiac imaging included atria, atrial function was not used in the adjustment. Second, we used non-invasive imaging and could not assess blood flow within the pulmonary vascular system. Third, our findings generate hypotheses and require further confirmation in other designs and populations. Fourth, our study may represent a small sample that recruited a cohort of participants with European ancestry, and the generalizability may be limited to other geographic regions.

## Conclusion

In subjects free of cardiovascular diseases, RV and LV parameters were strongly associated, suggesting that RV function is crucial for LV function. Given the critical role of the RV in the pulmonary circulation including lung volumes, lung volumes did not modify LV function in subjects without cardiovascular diseases.

## Supplementary Information

Supplementary Information.

## Data Availability

The datasets used and/or analyzed during the current study are available from the corresponding author on reasonable request. Alternatively, for access to KORA study data, requests can be directed to kora.passt@helmholtz-muenchen.de and are subject to approval by the KORA Board.

## Abbreviations

BSA: Body surface area
cine-SSFP: Cine steady-state free precession
COPD: Chronic obstructive pulmonary disease
CT: Computed tomography
eGFR: Estimated glomerular filtration rate
FOV: Field of view
HOMA-IR: Homeostasis model assessment-insulin resistance index
IGT: Impaired glucose tolerance
IQRs: Interquartile ranges
KORA: Cooperative Health Research in the Region of Augsburg
LV: Left ventricle
LVEF: Left ventricle ejection fraction
MRI: Magnetic resonance imaging
OGTT: Oral glucose tolerance test
RV: Right ventricle
SD: Standard deviation
TE: Echo time
TR: Repetition time
