# Supplementary material for: Relationship between right and left ventricle function in subjects free of cardiovascular diseases: a population-based MRI study
Source: Sci Rep. 2026 Jan 29;16:4110. doi: 10.1038/s41598-025-30588-z (PMC12858920; doi:10.1038/s41598-025-30588-z)
Supplement: Supplementary file 1 — Supplementary Information. [file 41598_2025_30588_MOESM1_ESM.pdf]

Supplementary Material. Relationship between Right and Left Ventricle Function in Subjects Free of Cardiovascular Diseases: A Population-Based MRI Study

Ricarda von Krüchten[1], Roberto Lorbeer[2], Susanne Rospleszcz[1], Annette Peters[3,4,5], Stefan Karrasch[4,6,7], Holger Schulz[5,7], Bernard E. Bulwer[8], Charlotte Wintergerst[1], Esther Askani[1], Thierno Diallo[1], Fabian Bamberg[1], Christopher L. Schlett[1], Blerim Mujaj*[1,9,]

Affiliations:

1. Department of Diagnostic and Interventional Radiology, Medical Center, Faculty of Medicine, University of Freiburg, Hugstetter Straße 55, 79106 Freiburg, Germany
2. Department of Radiology, University Hospital of Munich, Ludwig-Maximilians-University, Marchioninistraße 15, 81377 Munich, Germany
3. Chair of Epidemiology, Institute for Medical Information Processing, Biometry and Epidemiology, Medical Faculty, Ludwig-Maximilians-University Munich, Marchioninistr. 15, 81377 Munich, Germany
4. Institute of Epidemiology, Helmholtz Zentrum München - German Research Center for Environmental Health, Ingolstädter Landstraße 1, 85764 Neuherberg, Germany
5. German Center for Diabetes Research (DZD), partner site Neuherberg, Ingolstädter Landstraße 1, 85764 Neuherberg, Germany
6. Institute and Outpatient Clinic for Occupational, Social and Environmental Medicine, University Hospital of Munich, Ludwig-Maximilians-University, Ziemssenstraße 1, 80336 Munich, Germany
7. Comprehensive Pneumology Center Munich (CPC-M), Member of the German Center for Lung Research, Max-Lebsche-Platz 31, 81377 München, Germany
8. Noninvasive Cardiology - Echocardiography Department, Cardiovascular Division, Brigham and Women's Hospital, Boston, MA, USA
9. General Practice, Huisartsenpraktijk, Bremtstraat 116, 9320, Aalst, Belgium

Table S1 Right Ventricle and Left Ventricle Function Parameters according to tertiles of Lung Volumes

	Low tertile	Middle tertile	High tertile	P
**N = 361**	**121**	**120**	**120**	
	**(1.74-3.44L)**	**(3.45-4.35L)**	**(4.36-8.32L)**	
**Cardiac parameters**				
RV End-diastolic Volume, (mL)	156.4 (±35.2)	166.1 (±41.9)	174.2 (±39.9)	0.002
RV End-systolic Volume, (mL)	72.5 (±23.1)	79.1 (±26.6)	87.0 (±25.7)	<0.001
RV Stroke Volume, (mL)	84.0 (±17.2)	87.2 (±20.2)	87.2 (±20.9)	0.349
RV Ejection fraction, (%)	54.3 (±6.6)	53 (±6.7)	50.4 (±7.3)	<0.001
LV End-diastolic Volume, (mL)	127.1 (±30.1)	131.8 (±34.3)	133.0 (±33.0)	0.328
LV End-systolic Volume, (mL)	39.7 (±18.1)	41.0 (±18.0)	42.9 (±18.3)	0.406
LV Stroke Volume, (mL)	87.3 (±18.0)	90.8 (±20.8)	90.2 (±21.5)	0.367
LV Ejection fraction, (%)	69.5 (±7.4)	69.7 (±7.3)	68.5 (±8.7)	0.454
LV Peak ejection rate, (mL/s)	353.5 (±119.5)	358.1 (±141.2)	362.0 (±140.2)	0.884
LV Early diastolic filling rate, (mL/s)	242.2 (±107.1)	232.9 (±122.9)	216 (±115.6)	0.205
LV Late diastolic filling rate, (mL/s)	246.2 (±139.4)	251.6 (±153.0)	224.0 (±129.1)	0.277
LV Mass, diastolic, g	126.0 (±30.9)	140.7 (±32.9)	156.2 (±33.3)	<0.001

The values represent mean ± standard deviation (SD). P = p-value for difference (one-way ANOVA); Abbreviation: RV = right ventricle; LV = left ventricle

Table S2 Association between Lung volumes with Right and Left Ventricle parameters in participants without COPD (N=344)

Per SD	Model 1	p-value	Model 2	p-value	Model 3	p-value
	**RV End–diastolic Volume**					
Lung volumes	-3.36 (-7.66; 0.94)	0.125	-1.03 (-5.44; 3.39)	0.648	-2.29 (-6.63; 2.05)	0.299
	**RV End–systolic Volume**					
Lung volumes	-1.18 (-3.97; 1.61)	0.407	0.20 (-2.75; 3.14)	0.896	-0.60 (-3.51; 2.31)	0.686
	**RV Stroke Volume**					
Lung volumes	-2.17 (-4.44; 0.11)	0.062	-1.23 (-3.54; 1.09)	0.297	-1.70 (-3.99; 0.58)	0.144
	**RV Ejection fraction**					
Lung volumes	-0.30 (-1.12; 0.52)	0.471	-0.50 (-1.38; 0.38)	0.267	-0.37 (-1.24; 0.51)	0.413
	**LV End–diastolic Volume**					
Lung volumes	-4.28 (-7.94; -0.62)	0.022	-2.46 (-6.20; 1.29)	0.198	-3.48 (-7.13; 0.17)	0.062
	**LV End–systolic Volume**					
Lung volumes	-1.71 (-3.8; 0.39)	0.111	-0.6 (-2.82; 1.62)	0.594	-1.12 (-3.29; 1.06)	0.315
	**LV Stroke Volume**					
Lung volumes	-2.58 (-4.91; -0.25)	0.03	-1.85 (-4.20; 0.49)	0.120	-2.36 (-4.67; -0.06)	0.044
	**LV Ejection fraction**					
Lung volumes	0.17 (-0.77; 1.12)	0.719	-0.18 (-1.18; 0.83)	0.730	-0.04 (-1.04; 0.95)	0.93
	**LV Peak ejection rate**					
Lung volumes	-7.15 (-23.38; 9.08)	0.387	-7.08 (-23.7; 9.54)	0.403	-10.16 (-26.61; 6.3)	0.226
	**LV Early diastolic rate**					
Lung volumes	-13.41 (-26.96;0.15)	0.053	-15.75 (-29.4; -2.11)	0.024	-18.12 (-31.72; -4.53)	0.009
	**LV Late diastolic rate**					
Lung volumes	-18.38 (-35.79; -0.98)	0.039	-15.86 (-34.46; 2.74)	0.094	-17.69 (-35.8; 0.42)	0.055
	**LV Mass**					
Lung volumes	1.29 (-1.9; 4.48)	0.427	3.91 (0.92; 6.91)	0.011	3.51 (0.53; 6.49)	0.021

The beta estimate given with a 95% confidence interval represents the estimate size between lung volumes and cardiac right and left ventricle from linear regression model. The model 1 = adjusted for sex and age; model 2 = model 1 + smoking, alcohol use, BMI, systolic blood pressure, diastolic blood pressure, diabetes mellitus, total cholesterol and eGFR; model 3 = model 2 + insulin, glucose, antihypertensive medication, lipid lowering medication; CI = 95% confidence interval; SD = standard deviation. Abbreviation: BMI = body mass index; eGFR = estimated glomerular filtration rate.

Table S3 Association between Right Ventricle function parameters and Left Ventricle function parameters, according to tertiles of Lung Volumes

Per SD	Model 1	p-value	Model 2	p-value	Model 3	p-value
Low tertile n=121			**LV End–diastolic Volume**			
RV End-diastolic volume	30.55 (26.48; 34.62)	<0.001	30.51 (25.98; 35.03)	<0.001	29.64 (24.99; 34.28)	<0.001
			**LV End–systolic Volume**			
RV End-diastolic volume	12.84 (9.13; 16.55)	<0.001	13.56 (9.53; 17.59)	<0.001	12.04 (8.11; 15.98)	<0.001
			**LV Stroke Volume**			
RV End-diastolic volume	17.68 (15.05; 20.31)	<0.001	16.94 (13.99; 19.88)	<0.001	17.6 (14.48; 20.72)	<0.001
			**LV Ejection fraction**			
RV End-diastolic volume	-1.99 (-3.81; -0.18)	0.032	-2.52 (-4.54; -0.5)	0.015	-1.71 (-3.73; 0.32)	0.098
			**LV Peak ejection rate**			
RV End-diastolic volume	84.59 (60.46; 108.73)	<0.001	97.14 (70.59; 123.68)	<0.001	97.68 (69.21; 126.15)	<0.001
			**LV Early diastolic rate**			
RV End-diastolic volume	77.13 (56.67; 97.59)	<0.001	87.46 (64.96; 109.96)	<0.001	91.35 (67.16; 115.55)	<0.001
			**LV Late diastolic rate**			
RV End-diastolic volume	34.31 (-0.19; 68.8)	0.051	25.72 (-12.8; 64.24)	0.188	28.33 (-11.25; 67.91)	0.159
			**LV Mass**			
RV End-diastolic volume	16.33 (10.73; 21.93)	<0.001	12.98 (7.82; 18.15)	<0.001	10.88 (5.64; 16.12)	<0.001

The beta estimate given with a 95% confidence interval represents the estimate size between cardiac right and left ventricle, according to tertiles of lung volumes, from linear regression model. The model 1 = adjusted for sex and age; model 2 = model 1 + smoking, alcohol use, BMI, systolic blood pressure, diastolic blood pressure, diabetes mellitus, total cholesterol and eGFR; model 3 = model 2 + insulin, glucose, antihypertensive medication, lipid lowering medication; CI = 95% confidence interval; SD = standard deviation. Abbreviation: BMI = body mass index; eGFR = estimated glomerular filtration rate.

Table S4 Association between Right Ventricle function parameters and Left Ventricle function parameters, according to tertiles of Lung Volumes

**Per SD**	**Model 1**	**p-value**	**Model 2**	**p-value**	**Model 3**	**p-value**
Low tertile n=121			**LV End–diastolic Volume**			
RV End-systolic volume	23.74 (18.1; 29.39)	<0.001	21.6 (15.59; 27.62)	<0.001	19.65 (13.38; 25.92)	<0.001
			**LV End–systolic Volume**			
RV End-systolic volume	14.28 (10.58; 17.97)	<0.001	14.25 (10.38; 18.12)	<0.001	12.46 (8.64; 16.28)	<0.001
			**LV Stroke Volume**			
RV End-systolic volume	9.45 (5.52; 13.38)	<0.001	7.36 (3.26; 11.45)	0.001	7.2 (2.81; 11.58)	0.002
			**LV Ejection fraction**			
RV End-systolic volume	-4.32 (-6.05; -2.58)	<0.001	-4.78 (-6.62; -2.95)	<0.001	-4.08 (-5.94; -2.21)	<0.001
			**LV Peak ejection rate**			
RV End-systolic volume	55.81 (28.1; 83.51)	<0.001	59.99 (30.09; 89.89)	<0.001	56.68 (24.61; 88.75)	0.001
			**LV Early diastolic rate**			
RV End-systolic volume	50.37 (26.5; 74.25)	<0.001	52.08 (26.22; 77.94)	<0.001	52.18 (24.21; 80.15)	<0.001
			**LV Late diastolic rate**			
RV End-systolic volume	-14.46 (-50.46; 21.55)	0.428	-32.15 (-70.02; 5.71)	0.095	-34.12 (-73.07; 4.84)	0.085
			**LV Mass**			
RV End-systolic volume	11.87 (5.71; 18.03)	<0.001	8.41 (2.98; 13.84)	0.003	5.88 (0.4; 11.36)	0.036

The beta estimate given with a 95% confidence interval represents the estimate size between cardiac right and left ventricle, according to tertiles of lung volumes, from linear regression model. The model 1 = adjusted for sex and age; model 2 = model 1 + smoking, alcohol use, BMI, systolic blood pressure, diastolic blood pressure, diabetes mellitus, total cholesterol and eGFR; model 3 = model 2 + insulin, glucose, antihypertensive medication, lipid lowering medication; CI = 95% confidence interval; SD = standard deviation. Abbreviation: BMI = body mass index; eGFR = estimated glomerular filtration rate.

Table S5 Association between Right Ventricle function parameters and Left Ventricle function parameters, according to tertiles of Lung Volumes

Per SD	Model 1	p-value	Model 2	p-value	Model 3	p-value
Low tertile n=121			**LV End–diastolic Volume**			
RV Stroke volume	24.64 (20.62; 28.67)	<0.001	23.75 (19.38; 28.12)	<0.001	23.11 (18.93; 27.28)	<0.001
			**LV End–systolic Volume**			
RV Stroke volume	6.28 (2.62; 9.95)	0.001	5.99 (2.06; 9.92)	0.003	5.29 (1.64; 8.95)	0.005
			**LV Stroke Volume**			
RV Stroke volume	18.34 (16.91; 19.76)	<0.001	17.75 (16.19; 19.3)	<0.001	17.82 (16.19; 19.44)	<0.001
			**LV Ejection fraction**			
RV Stroke volume	1.01 (-0.6; 2.62)	0.216	0.99 (-0.77; 2.75)	0.268	1.36 (-0.32; 3.04)	0.11
			**LV Peak ejection rate**			
RV Stroke volume	77.48 (56.81; 98.15)	<0.001	84 (61.48; 106.52)	<0.001	83.18 (59.9; 106.45)	<0.001
			**LV Early diastolic rate**			
RV Stroke volume	71.14 (53.75; 88.53)	<0.001	77.55 (58.77; 96.34)	<0.001	78.52 (58.91; 98.13)	<0.001
			**LV Late diastolic rate**			
RV Stroke volume	66.66 (38.55; 94.78)	<0.001	69.64 (39.29; 99.99)	<0.001	70.78 (40.73; 100.82)	<0.001
			**LV Mass**			
RV Stroke volume	13.88 (8.96; 18.81)	<0.001	10.86 (6.43; 15.29)	<0.001	9.73 (5.45; 14.01)	<0.001

The beta estimate given with a 95% confidence interval represents the estimate size between cardiac right and left ventricle, according to tertiles of lung volumes, from linear regression model. The model 1 = adjusted for sex and age; model 2 = model 1 + smoking, alcohol use, BMI, systolic blood pressure, diastolic blood pressure, diabetes mellitus, total cholesterol and eGFR; model 3 = model 2 + insulin, glucose, antihypertensive medication, lipid lowering medication; CI = 95% confidence interval; SD = standard deviation. Abbreviation: BMI = body mass index; eGFR = estimated glomerular filtration rate.

Table S6 Association between Right Ventricle function parameters and Left Ventricle function parameters, according to tertiles of Lung Volumes

**Per SD**	**Model 1**	**p-value**	**Model 2**	**p-value**	**Model 3**	**p-value**
Low tertile n=121			**LV End–diastolic Volume**			
RV Ejection fraction	-1.24 (-7.12; 4.64)	0.677	-0.86 (-6.58; 4.86)	0.766	1.41 (-4.21; 7.02)	0.62
			**LV End–systolic Volume**			
RV Ejection fraction	-6.63 (-10.17; -3.1)	<0.001	-6.35 (-9.85; -2.84)	<0.001	-4.76 (-8.09; -1.42)	0.006
			**LV Stroke Volume**			
RV Ejection fraction	5.36 (1.95; 8.77)	0.002	5.45 (2.23; 8.68)	0.001	6.13 (2.83; 9.44)	<0.001
			**LV Ejection fraction**			
RV Ejection fraction	4.12 (2.74; 5.51)	<0.001	4.03 (2.62; 5.44)	<0.001	3.5 (2.11; 4.89)	<0.001
			**LV Peak ejection rate**			
RV Ejection fraction	11.44 (-12.86; 35.75)	0.353	9.81 (-15.22; 34.83)	0.439	14.72 (-11.03; 40.48)	0.26
			**LV Early diastolic rate**			
RV Ejection fraction	13.65 (-7.34; 34.65)	0.2	13.38 (-8.19; 34.94)	0.221	17.12 (-5.37; 39.61)	0.134
			**LV Late diastolic rate**			
RV Ejection fraction	62.71 (35.18; 90.24)	<0.001	70.56 (43.72; 97.4)	<0.001	70.65 (43.86; 97.45)	<0.001
			**LV Mass**			
RV Ejection fraction	0.24 (-5.16; 5.64)	0.93	0.44 (-3.99; 4.88)	0.843	1.86 (-2.4; 6.12)	0.388

The beta estimate given with a 95% confidence interval represents the estimate size between cardiac right and left ventricle, according to tertiles of lung volumes, from linear regression model. The model 1 = adjusted for sex and age; model 2 = model 1 + smoking, alcohol use, BMI, systolic blood pressure, diastolic blood pressure,diabetes mellitus, total cholesterol and eGFR; model 3 = model 2 + insulin, glucose, antihypertensive medication, lipid lowering medication; CI = 95% confidence interval; SD = standard deviation. Abbreviation: BMI = body mass index; eGFR = estimated glomerular filtration rate.

Table S7 Association between Right Ventricle function parameters and Left Ventricle function parameters, according to tertiles of Lung Volumes

**Per SD**	**Model 1**	**p-value**	**Model 2**	**p-value**	**Model 3**	**p-value**
Middle tertile n=120			**LV End–diastolic Volume**			
RV End-diastolic volume	28.83 (25.72; 31.94)	<0.001	27.37 (23.91; 30.84)	<0.001	26.18 (22.25; 30.1)	<0.001
			**LV End–systolic Volume**			
RV End-diastolic volume	11.63 (9.17; 14.1)	<0.001	10.95 (8.14; 13.75)	<0.001	9.73 (6.55; 12.9)	<0.001
			**LV Stroke Volume**			
RV End-diastolic volume	17.15 (15; 19.3)	<0.001	16.37 (13.95; 18.79)	<0.001	16.38 (13.69; 19.06)	<0.001
			**LV Ejection fraction**			
RV End-diastolic volume	-1.79 (-3.13; -0.44)	0.01	-1.75 (-3.28; -0.23)	0.025	-1.15 (-2.86; 0.56)	0.185
			**LV Peak ejection rate**			
RV End-diastolic volume	110.83 (92.07; 129.59)	<0.001	107.82 (87.43; 128.21)	<0.001	107.14 (83.7; 130.58)	<0.001
			**LV Early diastolic rate**			
RV End-diastolic volume	89.59 (72.77; 106.41)	<0.001	86.85 (69.22; 104.49)	<0.001	90.59 (70.65; 110.53)	<0.001
			**LV Late diastolic rate**			
RV End-diastolic volume	39.97 (12.46; 67.48)	0.005	42.19 (11.75; 72.63)	0.007	40.59 (9.43; 71.74)	0.011
			**LV Mass**			
RV End-diastolic volume	8.97 (4.37; 13.58)	<0.001	8.92 (4.45; 13.39)	<0.001	9.47 (4.31; 14.63)	<0.001

The beta estimate given with a 95% confidence interval represents the estimate size between cardiac right and left ventricle, according to tertiles of lung volumes, from linear regression model. The model 1 = adjusted for sex and age; model 2 = model 1 + smoking, alcohol use, BMI, systolic blood pressure, diastolic blood pressure,diabetes mellitus, total cholesterol and eGFR; model 3 = model 2 + insulin, glucose, antihypertensive medication, lipid lowering medication; CI = 95% confidence interval; SD = standard deviation. Abbreviation: BMI = body mass index; eGFR = estimated glomerular filtration rate.

Table S8 Association between Right Ventricle function parameters and Left Ventricle function parameters, according to tertiles of Lung Volumes

Per SD	Model 1	p-value	Model 2	p-value	Model 3	p-value
Middle tertile n=120			**LV End–diastolic Volume**			
RV End-systolic volume	23.75 (19.17; 28.34)	<0.001	21.32 (16.6; 26.03)	<0.001	19.27 (14.13; 24.41)	<0.001
			**LV End–systolic Volume**			
RV End-systolic volume	12.12 (9.61; 14.64)	<0.001	11.31 (8.59; 14.02)	<0.001	10.26 (7.22; 13.3)	<0.001
			**LV Stroke Volume**			
RV End-systolic volume	11.6 (8.3; 14.91)	<0.001	9.98 (6.54; 13.42)	<0.001	8.97 (5.22; 12.72)	<0.001
			**LV Ejection fraction**			
RV End-systolic volume	-3.19 (-4.49; -1.89)	<0.001	-3.24 (-4.66; -1.83)	<0.001	-2.94 (-4.54; -1.35)	<0.001
			**LV Peak ejection rate**			
RV End-systolic volume	85.34 (61.52; 109.17)	<0.001	76.5 (51.69; 101.31)	<0.001	69.65 (41.75; 97.56)	<0.001
			**LV Early diastolic rate**			
RV End-systolic volume	64.55 (43.41; 85.69)	<0.001	56.77 (35.29; 78.24)	<0.001	54.74 (30.67; 78.82)	<0.001
			**LV Late diastolic rate**			
RV End-systolic volume	20.23 (-8.86; 49.32)	0.171	16.63 (-14.44; 47.7)	0.291	10.53 (-21.07; 42.12)	0.51
			**LV Mass**			
RV End-systolic volume	6.64 (1.75; 11.52)	0.008	6.71 (2.14; 11.28)	0.004	6.66 (1.42; 11.9)	0.013

The beta estimate given with a 95% confidence interval represents the estimate size between cardiac right and left ventricle, according to tertiles of lung volumes, from linear regression model. The model 1 = adjusted for sex and age; model 2 = model 1 + smoking, alcohol use, BMI, systolic blood pressure, diastolic blood pressure,diabetes mellitus, total cholesterol and eGFR; model 3 = model 2 + insulin, glucose, antihypertensive medication, lipid lowering medication; CI = 95% confidence interval; SD = standard deviation. Abbreviation: BMI = body mass index; eGFR = estimated glomerular filtration rate.

Table S9 Association between Right Ventricle function parameters and Left Ventricle function parameters, according to tertiles of Lung Volumes

Per SD	Model 1	p-value	Model 2	p-value	Model 3	p-value
Middle tertile n=120	**LV End–diastolic Volume**					
RV Stroke volume	26.7 (23.41; 29.99)	<0.001	25.21 (21.48; 28.94)	<0.001	23.21 (19.25; 27.18)	<0.001
	**LV End–systolic Volume**					
RV Stroke volume	7.83 (5.04; 10.62)	<0.001	6.54 (3.37; 9.7)	<0.001	4.9 (1.57; 8.23)	0.004
	**LV Stroke Volume**					
RV Stroke volume	18.82 (17.59; 20.05)	<0.001	18.61 (17.18; 20.05)	<0.001	18.24 (16.69; 19.78)	<0.001
	**LV Ejection fraction**					
RV Stroke volume	0.34 (-0.99; 1.67)	0.609	0.78 (-0.73; 2.29)	0.307	1.5 (-0.08; 3.08)	0.063
	**LV Peak ejection rate**					
RV Stroke volume	109.89 (92.52; 127.25)	<0.001	109.19 (90.32; 128.06)	<0.001	106.55 (85.91; 127.19)	<0.001
	**LV Early diastolic rate**					
RV Stroke volume	93.81 (79.14; 108.48)	<0.001	93.94 (78.79; 109.09)	<0.001	94.82 (78.26; 111.38)	<0.001
	**LV Late diastolic rate**					
RV Stroke volume	50.83 (25.09; 76.57)	<0.001	58.39 (29.95; 86.84)	<0.001	58.32 (30.6; 86.05)	<0.001
	**LV Mass**					
RV Stroke volume	9.14 (4.74; 13.53)	<0.001	8.49 (4.14; 12.85)	<0.001	8.75 (3.93; 13.57)	<0.001

The beta estimate given with a 95% confidence interval represents the estimate size between cardiac right and left ventricle, according to tertiles of lung volumes, from linear regression model. The model 1 = adjusted for sex and age; model 2 = model 1 + smoking, alcohol use, BMI, systolic blood pressure, diastolic blood pressure, diabetes mellitus, total cholesterol and eGFR; model 3 = model 2 + insulin, glucose, antihypertensive medication, lipid lowering medication; CI = 95% confidence interval; SD = standard deviation. Abbreviation: BMI = body mass index; eGFR = estimated glomerular filtration rate.

Table S10 Association between Right Ventricle function parameters and Left Ventricle function parameters, according to tertiles of Lung Volumes

Per SD	Model 1	p-value	Model 2	p-value	Model 3	p-value
Middle tertile n=120			**LV End–diastolic Volume**			
RV Ejection fraction	-2.37 (-8.78; 4.05)	0.466	-3.33 (-9.42; 2.76)	0.281	-1.88 (-7.83; 4.07)	0.532
			**LV End–systolic Volume**			
RV Ejection fraction	-6.31 (-9.52; -3.1)	<0.001	-6.77 (-9.91; -3.62)	<0.001	-6.2 (-9.37; -3.02)	<0.001
			**LV Stroke Volume**			
RV Ejection fraction	3.93 (0; 7.86)	0.05	3.43 (-0.37; 7.23)	0.077	4.31 (0.54; 8.07)	0.025
			**LV Ejection fraction**			
RV Ejection fraction	3.99 (2.75; 5.24)	<0.001	4.07 (2.77; 5.37)	<0.001	4.03 (2.67; 5.39)	<0.001
			**LV Peak ejection rate**			
RV Ejection fraction	10.31 (-18.61; 39.23)	0.481	7.7 (-20.52; 35.92)	0.59	16.43 (-12.38; 45.24)	0.261
			**LV Early diastolic rate**			
RV Ejection fraction	14.69 (-9.79; 39.17)	0.237	13.52 (-9.94; 36.97)	0.256	18.5 (-5.82; 42.82)	0.134
			**LV Late diastolic rate**			
RV Ejection fraction	17.49 (-12.12; 47.11)	0.244	20.77 (-9.57; 51.1)	0.178	24.5 (-4.58; 53.58)	0.098
			**LV Mass**			
RV Ejection fraction	1.64 (-3.47; 6.74)	0.527	0.25 (-4.41; 4.9)	0.917	1.04 (-3.99; 6.07)	0.682

The beta estimate given with a 95% confidence interval represents the estimate size between cardiac right and left ventricle, according to tertiles of lung volumes, from linear regression model. The model 1 = adjusted for sex and age; model 2 = model 1 + smoking, alcohol use, BMI, systolic blood pressure, diastolic blood pressure, diabetes mellitus, total cholesterol and eGFR; model 3 = model 2 + insulin, glucose, antihypertensive medication, lipid lowering medication; CI = 95% confidence interval; SD = standard deviation. Abbreviation: BMI = body mass index; eGFR = estimated glomerular filtration rate.

Table S11 Association between Right Ventricle function parameters and Left Ventricle function parameters, according to tertiles of Lung Volumes

Per SD	Model 1	p-value	Model 2	p-value	Model 3	p-value
High tertile n=120			**LV End–diastolic Volume**			
RV End-diastolic volume	25.83 (22.06; 29.59)	<0.001	24.31 (20.32; 28.3)	<0.001	24.38 (20.31; 28.44)	<0.001
			**LV End–systolic Volume**			
RV End-diastolic volume	9.19 (6.22; 12.16)	<0.001	8.8 (5.6; 12.01)	<0.001	9.12 (5.85; 12.38)	<0.001
			**LV Stroke Volume**			
RV End-diastolic volume	16.71 (13.98; 19.45)	<0.001	15.57 (12.8; 18.33)	<0.001	15.32 (12.52; 18.13)	<0.001
			**LV Ejection fraction**			
RV End-diastolic volume	-0.69 (-2.35; 0.98)	0.415	-0.71 (-2.48; 1.05)	0.425	-0.98 (-2.77; 0.81)	0.28
			**LV Peak ejection rate**			
RV End-diastolic volume	105.13 (85.41; 124.85)	<0.001	96.5 (76.13; 116.87)	<0.001	94 (74.08; 113.91)	<0.001
			**LV Early diastolic rate**			
RV End-diastolic volume	83.09 (67.15; 99.04)	<0.001	76.14 (59.99; 92.3)	<0.001	74.81 (58.8; 90.81)	<0.001
			**LV Late diastolic rate**			
RV End-diastolic volume	31.94 (6.86; 57.02)	0.013	25.45 (-1.11; 52.01)	0.06	23.81 (-3.41; 51.03)	0.086
			**LV Mass**			
RV End-diastolic volume	4.15 (-1.33; 9.64)	0.137	5.23 (0; 10.47)	0.05	5.94 (0.66; 11.22)	0.028

The beta estimate given with a 95% confidence interval represents the estimate size between cardiac right and left ventricle, according to tertiles of lung volumes, from linear regression model. The model 1 = adjusted for sex and age; model 2 = model 1 + smoking, alcohol use, BMI, systolic blood pressure, diastolic blood pressure, diabetes mellitus, total cholesterol and eGFR; model 3 = model 2 + insulin, glucose, antihypertensive medication, lipid lowering medication; CI = 95% confidence interval; SD = standard deviation. Abbreviation: BMI = body mass index; eGFR = estimated glomerular filtration rate.

Table S12 Association between Right Ventricle function parameters and Left Ventricle function parameters, according to tertiles of Lung Volumes

Per SD	Model 1	p-value	Model 2	p-value	Model 3	p-value
High tertile n=120			**LV End–diastolic Volume**			
RV End-systolic volume	17.56 (12.6; 22.51)	<0.001	16.26 (11.29; 21.24)	<0.001	16.26 (11.23; 21.29)	<0.001
			**LV End–systolic Volume**			
RV End-systolic volume	9.54 (6.7; 12.38)	<0.001	9.02 (6.02; 12.02)	<0.001	9.13 (6.09; 12.18)	<0.001
			**LV Stroke Volume**			
RV End-systolic volume	8.11 (4.38; 11.84)	<0.001	7.31 (3.68; 10.95)	<0.001	7.2 (3.57; 10.84)	<0.001
			**LV Ejection fraction**			
RV End-systolic volume	-2.87 (-4.41; -1.32)	<0.001	-2.76 (-4.37; -1.15)	0.001	-2.88 (-4.49; -1.27)	0.001
			**LV Peak ejection rate**			
RV End-systolic volume	66.67 (42.66; 90.68)	<0.001	57.66 (33.75; 81.56)	<0.001	57.22 (34.05; 80.4)	<0.001
			**LV Early diastolic rate**			
RV End-systolic volume	56.57 (37.69; 75.46)	<0.001	48.34 (29.68; 67.01)	<0.001	48.18 (29.87; 66.49)	<0.001
			**LV Late diastolic rate**			
RV End-systolic volume	9.54 (-15.55; 34.62)	0.453	4.63 (-21.17; 30.44)	0.723	3.95 (-22.24; 30.13)	0.766
			**LV Mass**			
RV End-systolic volume	0.82 (-4.58; 6.23)	0.763	1.97 (-3.12; 7.05)	0.445	2.32 (-2.79; 7.43)	0.37

The beta estimate given with a 95% confidence interval represents the estimate size between cardiac right and left ventricle, according to tertiles of lung volumes, from linear regression model. The model 1 = adjusted for sex and age; model 2 = model 1 + smoking, alcohol use, BMI, systolic blood pressure, diastolic blood pressure, diabetes mellitus, total cholesterol and eGFR; model 3 = model 2 + insulin, glucose, antihypertensive medication, lipid lowering medication; CI = 95% confidence interval; SD = standard deviation. Abbreviation: BMI = body mass index; eGFR = estimated glomerular filtration rate.

Table S13 Association between Right Ventricle function parameters and Left Ventricle function parameters, according to tertiles of Lung Volumes

Per SD	Model 1	p-value	Model 2	p-value	Model 3	p-value
High tertile n=120			**LV End–diastolic Volume**			
RV Stroke volume	23.94 (20.49; 27.39)	<0.001	22.76 (19.01; 26.52)	<0.001	23.02 (19.14; 26.91)	<0.001
			**LV End–systolic Volume**			
RV Stroke volume	4.61 (1.57; 7.65)	0.003	4.24 (0.94; 7.55)	0.012	4.57 (1.15; 7.99)	0.009
			**LV Stroke Volume**			
RV Stroke volume	19.35 (18.03; 20.68)	<0.001	18.54 (17.16; 19.93)	<0.001	18.48 (17.05; 19.92)	<0.001
			**LV Ejection fraction**			
RV Stroke volume	2.22 (0.73; 3.71)	0.004	2.26 (0.65; 3.86)	0.006	2.03 (0.37; 3.69)	0.017
			**LV Peak ejection rate**			
RV Stroke volume	102.99 (85.92; 120.06)	<0.001	98.96 (81.44; 116.48)	<0.001	95.75 (78.14; 113.36)	<0.001
			**LV Early diastolic rate**			
RV Stroke volume	77.07 (62.42; 91.73)	<0.001	74.74 (60.16; 89.31)	<0.001	73.01 (58.2; 87.82)	<0.001
			**LV Late diastolic rate**			
RV Stroke volume	44.61 (22.29; 66.93)	<0.001	40.11 (15.96; 64.26)	0.001	38.79 (13.72; 63.86)	0.003
			**LV Mass**			
RV Stroke volume	6.2 (1.21; 11.18)	0.015	6.91 (2.08; 11.74)	0.005	7.82 (2.92; 12.71)	0.002

The beta estimate given with a 95% confidence interval represents the estimate size between cardiac right and left ventricle, according to tertiles of lung volumes, from linear regression model. The model 1 = adjusted for sex and age; model 2 = model 1 + smoking, alcohol use, BMI, systolic blood pressure, diastolic blood pressure, diabetes mellitus, total cholesterol and eGFR; model 3 = model 2 + insulin, glucose, antihypertensive medication, lipid lowering medication; CI = 95% confidence interval; SD = standard deviation. Abbreviation: BMI = body mass index; eGFR = estimated glomerular filtration rate.

Table S14 Association between Right Ventricle function parameters and Left Ventricle function parameters, according to tertiles of Lung Volumes

Per SD	Model 1	p-value	Model 2	p-value	Model 3	p-value
High tertile n=120			**LV End–diastolic Volume**			
RV Ejection fraction	2.71 (-2.67; 8.09)	0.321	1.69 (-3.65; 7.03)	0.531	1.21 (-4.26; 6.69)	0.661
			**LV End–systolic Volume**			
RV Ejection fraction	-4.79 (-7.71; -1.87)	0.002	-4.88 (-7.89; -1.87)	0.002	-4.95 (-8.05; -1.86)	0.002
			**LV Stroke Volume**			
RV Ejection fraction	7.44 (4.04; 10.85)	<0.001	6.53 (3.21; 9.85)	<0.001	6.14 (2.76; 9.51)	<0.001
			**LV Ejection fraction**			
RV Ejection fraction	4.28 (3.01; 5.54)	<0.001	4.13 (2.8; 5.45)	<0.001	4.07 (2.72; 5.42)	<0.001
			**LV Peak ejection rate**			
RV Ejection fraction	20.64 (-3.68; 44.96)	0.096	19.79 (-3.89; 43.46)	0.101	14.45 (-9.05; 37.96)	0.225
			**LV Early diastolic rate**			
RV Ejection fraction	9.2 (-10.42; 28.81)	0.355	10.38 (-8.49; 29.25)	0.278	6.73 (-12.16; 25.63)	0.481
			**LV Late diastolic rate**			
RV Ejection fraction	22.52 (-0.06; 45.11)	0.051	20.03 (-3.14; 43.2)	0.09	18.93 (-4.86; 42.72)	0.118
			**LV Mass**			
RV Ejection fraction	3.48 (-1.42; 8.37)	0.162	2.64 (-1.97; 7.26)	0.258	2.81 (-1.88; 7.49)	0.237

The beta estimate given with a 95% confidence interval represents the estimate size between cardiac right and left ventricle, according to tertiles of lung volumes, from linear regression model. The model 1 = adjusted for sex and age; model 2 = model 1 + smoking, alcohol use, BMI, systolic blood pressure, diastolic blood pressure, diabetes mellitus, total cholesterol and eGFR; model 3 = model 2 + insulin, glucose, antihypertensive medication, lipid lowering medication; CI = 95% confidence interval; SD = standard deviation. Abbreviation: BMI = body mass index; eGFR = estimated glomerular filtration rate.

Table S15 Association between Right Ventricle function parameters and Left Ventricle function parameters, according to gender

Per SD	Model 1	p-value	Model 2	p-value	Model 3	p-value
Female n=154			**LV End–diastolic Volume**			
RV End-diastolic volume	26.99 (23.87; 30.11)	<0.001	26.67 (23.34; 30)	<0.001	26.52 (23.09; 29.96)	<0.001
			**LV End–systolic Volume**			
RV End-diastolic volume	9.83 (7.23; 12.42)	<0.001	10.35 (7.64; 13.07)	<0.001	9.6 (6.87; 12.32)	<0.001
			**LV Stroke Volume**			
RV End-diastolic volume	17.14 (15; 19.28)	<0.001	16.32 (13.97; 18.66)	<0.001	16.9 (14.45; 19.35)	<0.001
			**LV Ejection fraction**			
RV End-diastolic volume	-1.51 (-2.89; -0.13)	0.033	-1.77 (-3.26; -0.29)	0.02	-1.29 (-2.83; 0.25)	0.099
			**LV Peak ejection rate**			
RV End-diastolic volume	84.07 (66.11; 102.03)	<0.001	82.03 (62.45; 101.61)	<0.001	86.75 (65.93; 107.57)	<0.001
			**LV Early diastolic rate**			
RV End-diastolic volume	76.47 (59.37; 93.57)	<0.001	75.22 (57.3; 93.13)	<0.001	80.1 (60.85; 99.35)	<0.001
			**LV Late diastolic rate**			
RV End-diastolic volume	37.9 (10.85; 64.96)	0.006	39.45 (9.71; 69.18)	0.01	47.54 (16.84; 78.24)	0.003
			**LV Mass**			
RV End-diastolic volume	14.53 (10.04; 19.02)	<0.001	11.35 (7.43; 15.26)	<0.001	10.63 (6.5; 14.77)	<0.001

The beta estimate given with a 95% confidence interval represents the estimate size between cardiac right and left ventricle from linear regression model, according to gender. The model 1 = adjusted for age; model 2 = model 1 + smoking, alcohol use, BMI, systolic blood pressure, diastolic blood pressure, diabetes mellitus, total cholesterol and eGFR; model 3 = model 2 + insulin, glucose, antihypertensive medication, lipid lowering medication; CI = 95% confidence interval; SD = standard deviation. Abbreviation: BMI = body mass index; eGFR = estimated glomerular filtration rate.

Table S16 Association between Right Ventricle function parameters and Left Ventricle function parameters, according to gender

Per SD	Model 1	p-value	Model 2	p-value	Model 3	p-value
Female n=154	**LV End–diastolic Volume**					
RV End-systolic volume	20.56 (15.99; 25.12)	<0.001	18.81 (14.26; 23.37)	<0.001	18.09 (13.28; 22.91)	<0.001
	**LV End–systolic Volume**					
RV End- systolic volume	10.42 (7.69; 13.15)	<0.001	10.22 (7.51; 12.94)	<0.001	9.26 (6.48; 12.05)	<0.001
	**LV Stroke Volume**					
RV End- systolic volume	10.15 (6.86; 13.45)	<0.001	8.63 (5.35; 11.9)	<0.001	8.84 (5.35; 12.34)	<0.001
	**LV Ejection fraction**					
RV End- systolic volume	-3.23 (-4.61; -1.84)	<0.001	-3.38 (-4.78; -1.97)	<0.001	-2.95 (-4.44; -1.47)	<0.001
	**LV Peak ejection rate**					
RV End- systolic volume	56.92 (35.07; 78.77)	<0.001	50.85 (28.63; 73.08)	<0.001	53.18 (29.12; 77.25)	<0.001
	**LV Early diastolic rate**					
RV End- systolic volume	48.46 (27.68; 69.25)	<0.001	42.92 (22.35; 63.5)	<0.001	45.72 (23.28; 68.17)	<0.001
	**LV Late diastolic rate**					
RV End- systolic volume	-3.13 (-32.36; 26.1)	0.833	-6.24 (-36.56; 24.08)	0.685	1.48 (-30.5; 33.45)	0.927
	**LV Mass**					
RV End- systolic volume	10.22 (5.15; 15.3)	<0.001	7.53 (3.38; 11.68)	<0.001	6.46 (2.04; 10.87)	0.004

The beta estimate given with a 95% confidence interval represents the estimate size between cardiac right and left ventricle from linear regression model, according to gender. The model 1 = adjusted for age; model 2 = model 1 + smoking, alcohol use, BMI, systolic blood pressure, diastolic blood pressure, diabetes mellitus, total cholesterol and eGFR; model 3 = model 2 + insulin, glucose, antihypertensive medication, lipid lowering medication; CI = 95% confidence interval; SD = standard deviation. Abbreviation: BMI = body mass index; eGFR = estimated glomerular filtration rate.

Table S17 Association between Right Ventricle function parameters and Left Ventricle function parameters, according to gender

Per SD	Model 1	p-value	Model 2	p-value	Model 3	p-value
Female n=154			**LV End–diastolic Volume**			
RV Stroke volume	23.68 (20.88; 26.48)	<0.001	24.22 (21.16; 27.28)	<0.001	23.83 (20.86; 26.8)	<0.001
			**LV End–systolic Volume**			
RV Stroke volume	5.91 (3.39; 8.42)	<0.001	6.2 (3.45; 8.94)	<0.001	5.88 (3.25; 8.51)	<0.001
			**LV Stroke Volume**			
RV Stroke volume	17.72 (16.53; 18.9)	<0.001	17.98 (16.66; 19.3)	<0.001	17.89 (16.5; 19.28)	<0.001
			**LV Ejection fraction**			
RV Stroke volume	0.6 (-0.64; 1.83)	0.343	0.73 (-0.65; 2.1)	0.299	0.93 (-0.44; 2.3)	0.182
			**LV Peak ejection rate**			
RV Stroke volume	79.98 (64.82; 95.13)	<0.001	81.68 (64.68; 98.69)	<0.001	83.44 (65.83; 101.04)	<0.001
			**LV Early diastolic rate**			
RV Stroke volume	75.94 (61.88; 90)	<0.001	79.14 (64.17; 94.11)	<0.001	80.63 (64.87; 96.4)	<0.001
			**LV Late diastolic rate**			
RV Stroke volume	62.76 (40.41; 85.11)	<0.001	73.21 (48.25; 98.17)	<0.001	74.06 (48.91; 99.21)	<0.001
			**LV Mass**			
RV Stroke volume	13.48 (9.56; 17.39)	<0.001	10.84 (7.31; 14.37)	<0.001	10.4 (6.81; 13.98)	<0.001

The beta estimate given with a 95% confidence interval represents the estimate size between cardiac right and left ventricle from linear regression model, according to gender. The model 1 = adjusted for age; model 2 = model 1 + smoking, alcohol use, BMI, systolic blood pressure, diastolic blood pressure, diabetes mellitus, total cholesterol and eGFR; model 3 = model 2 + insulin, glucose, antihypertensive medication, lipid lowering medication; CI = 95% confidence interval; SD = standard deviation. Abbreviation: BMI = body mass index; eGFR = estimated glomerular filtration rate.

Table S18 Association between Right Ventricle function parameters and Left Ventricle function parameters, according to gender

Per SD	Model 1	p-value	Model 2	p-value	Model 3	p-value
Female n=154			**LV End–diastolic Volume**			
RV Ejection fraction	-0.42 (-4.99; 4.15)	0.856	-1.09 (-5.52; 3.33)	0.626	0.28 (-4.23; 4.78)	0.903
			**LV End–systolic Volume**			
RV Ejection fraction	-4.49 (-6.99; -1.99)	0.001	-4.53 (-6.99; -2.08)	<0.001	-3.61 (-6.06; -1.16)	0.004
			**LV Stroke Volume**			
RV Ejection fraction	4.02 (1.11; 6.94)	0.007	3.39 (0.58; 6.19)	0.018	3.84 (0.92; 6.77)	0.01
			**LV Ejection fraction**			
RV Ejection fraction	3.15 (2.06; 4.24)	<0.001	3.13 (2.03; 4.22)	<0.001	2.87 (1.74; 4.01)	<0.001
			**LV Peak ejection rate**			
RV Ejection fraction	11.85 (-7.28; 30.99)	0.223	8.86 (-10.12; 27.85)	0.358	11.65 (-8.51; 31.82)	0.255
			**LV Early diastolic rate**			
RV Ejection fraction	15.75 (-2.09; 33.59)	0.083	14.12 (-3.16; 31.4)	0.108	16.31 (-2.21; 34.83)	0.084
			**LV Late diastolic rate**			
RV Ejection fraction	50.87 (28.59; 73.15)	<0.001	51.99 (29.29; 74.68)	<0.001	48.14 (24.3; 71.98)	<0.001
			**LV Mass**			
RV Ejection fraction	1.24 (-3.08; 5.57)	0.571	0.28 (-3.19; 3.74)	0.875	1.31 (-2.26; 4.89)	0.469

The beta estimate given with a 95% confidence interval represents the estimate size between cardiac right and left ventricle from linear regression model, according to gender. The model 1 = adjusted for age; model 2 = model 1 + smoking, alcohol use, BMI, systolic blood pressure, diastolic blood pressure, diabetes mellitus, total cholesterol and eGFR; model 3 = model 2 + insulin, glucose, antihypertensive medication, lipid lowering medication; CI = 95% confidence interval; SD = standard deviation. Abbreviation: BMI = body mass index; eGFR = estimated glomerular filtration rate.

Table S19 Association between Right Ventricle function parameters and Left Ventricle function parameters, according to gender

Per SD	Model 1	p-value	Model 2	p-value	Model 3	p-value
Male n=207	**LV End–diastolic Volume**					
RV End-diastolic volume	28.65 (25.9; 31.4)	<0.001	27.44 (24.48; 30.4)	<0.001	27.22 (24.15; 30.28)	<0.001
	**LV End–systolic Volume**					
RV End-diastolic volume	11.66 (9.39; 13.92)	<0.001	11.35 (8.9; 13.8)	<0.001	11.25 (8.74; 13.76)	<0.001
	**LV Stroke Volume**					
RV End-diastolic volume	17.01 (15.12; 18.9)	<0.001	16.09 (14.08; 18.1)	<0.001	15.98 (13.9; 18.05)	<0.001
	**LV Ejection fraction**					
RV End-diastolic volume	-1.48 (-2.68; -0.27)	0.016	-1.61 (-2.9; -0.32)	0.015	-1.61 (-2.92; -0.3)	0.016
	**LV Peak ejection rate**					
RV End-diastolic volume	111.51 (96.25; 126.77)	<0.001	107.93 (92.08; 123.79)	<0.001	106.18 (89.93; 122.44)	<0.001
	**LV Early diastolic rate**					
RV End-diastolic volume	88.19 (75.88; 100.5)	<0.001	85.01 (72.55; 97.47)	<0.001	85.67 (72.94; 98.4)	<0.001
	**LV Late diastolic rate**					
RV End-diastolic volume	37.69 (17.07; 58.31)	<0.001	31.25 (8.93; 53.58)	0.006	25.19 (2.58; 47.8)	0.029
	**LV Mass**					
RV End-diastolic volume	6.01 (2.07; 9.95)	0.003	7.94 (4.09; 11.78)	<0.001	7.6 (3.65; 11.56)	<0.001

The beta estimate given with a 95% confidence interval represents the estimate size between cardiac right and left ventricle from linear regression model, according to gender. The model 1 = adjusted for age; model 2 = model 1 + smoking, alcohol use, BMI, systolic blood pressure, diastolic blood pressure, diabetes mellitus, total cholesterol and eGFR; model 3 = model 2 + insulin, glucose, antihypertensive medication, lipid lowering medication; CI = 95% confidence interval; SD = standard deviation. Abbreviation: BMI = body mass index; eGFR = estimated glomerular filtration rate.

Table S20 Association between Right Ventricle function parameters and Left Ventricle function parameters, according to gender

Per SD	Model 1	p-value	Model 2	p-value	Model 3	p-value
Male n=207			**LV End–diastolic Volume**			
RV End-systolic volume	21.53 (17.8; 25.27)	<0.001	19.56 (15.74; 23.37)	<0.001	18.97 (15.05; 22.89)	<0.001
			**LV End–systolic Volume**			
RV End- systolic volume	12.01 (9.81; 14.21)	<0.001	11.54 (9.24; 13.85)	<0.001	11.36 (8.99; 13.72)	<0.001
			**LV Stroke Volume**			
RV End- systolic volume	9.55 (6.87; 12.23)	<0.001	8.03 (5.33; 10.72)	<0.001	7.64 (4.87; 10.4)	<0.001
			**LV Ejection fraction**			
RV End- systolic volume	-3.33 (-4.45; -2.21)	<0.001	-3.52 (-4.68; -2.37)	<0.001	-3.53 (-4.71; -2.36)	<0.001
			**LV Peak ejection rate**			
RV End- systolic volume	78.15 (59.56; 96.73)	<0.001	69.36 (50.53; 88.2)	<0.001	66.57 (47.39; 85.76)	<0.001
			**LV Early diastolic rate**			
RV End- systolic volume	63.29 (48.52; 78.06)	<0.001	55.95 (41.23; 70.66)	<0.001	55.63 (40.59; 70.66)	<0.001
			**LV Late diastolic rate**			
RV End- systolic volume	14.89 (-6.09; 35.88)	0.163	7.26 (-14.66; 29.19)	0.514	2.3 (-19.73; 24.32)	0.837
			**LV Mass**			
RV End- systolic volume	3.31 (-0.66; 7.27)	0.102	4.67 (0.86; 8.48)	0.017	3.97 (0.06; 7.88)	0.047

The beta estimate given with a 95% confidence interval represents the estimate size between cardiac right and left ventricle from linear regression model, according to gender. The model 1 = adjusted for age; model 2 = model 1 + smoking, alcohol use, BMI, systolic blood pressure, diastolic blood pressure, diabetes mellitus, total cholesterol and eGFR; model 3 = model 2 + insulin, glucose, antihypertensive medication, lipid lowering medication; CI = 95% confidence interval; SD = standard deviation. Abbreviation: BMI = body mass index; eGFR = estimated glomerular filtration rate.

Table S21 Association between Right Ventricle function parameters and Left Ventricle function parameters, according to gender

Per SD	Model 1	p-value	Model 2	p-value	Model 3	p-value
Male n=207	**LV End–diastolic Volume**					
RV Stroke volume	26.02 (23.22; 28.81)	<0.001	24.6 (21.6; 27.59)	<0.001	24.36 (21.33; 27.4)	<0.001
	**LV End–systolic Volume**					
RV Stroke volume	6.67 (4.21; 9.13)	<0.001	5.81 (3.18; 8.44)	<0.001	5.67 (3.01; 8.33)	<0.001
	**LV Stroke Volume**					
RV Stroke volume	19.35 (18.36; 20.34)	<0.001	18.77 (17.73; 19.81)	<0.001	18.68 (17.61; 19.75)	<0.001
	**LV Ejection fraction**					
RV Stroke volume	1.34 (0.2; 2.48)	0.021	1.56 (0.35; 2.77)	0.012	1.58 (0.36; 2.8)	0.011
	**LV Peak ejection rate**					
RV Stroke volume	108.31 (94.31; 122.32)	<0.001	106.46 (92.26; 120.66)	<0.001	104.5 (90.12; 118.88)	<0.001
	**LV Early diastolic rate**					
RV Stroke volume	83.89 (72.34; 95.44)	<0.001	82.22 (70.83; 93.61)	<0.001	81.94 (70.36; 93.52)	<0.001
	**LV Late diastolic rate**					
RV Stroke volume	50.37 (31.51; 69.22)	<0.001	46.78 (26.43; 67.13)	<0.001	41.4 (20.88; 61.91)	<0.001
	**LV Mass**					
RV Stroke volume	6.87 (3.19; 10.56)	<0.001	8.35 (4.77; 11.93)	<0.001	8.44 (4.81; 12.08)	<0.001

The beta estimate given with a 95% confidence interval represents the estimate size between cardiac right and left ventricle from linear regression model, according to gender. The model 1 = adjusted for age; model 2 = model 1 + smoking, alcohol use, BMI, systolic blood pressure, diastolic blood pressure, diabetes mellitus, total cholesterol and eGFR; model 3 = model 2 + insulin, glucose, antihypertensive medication, lipid lowering medication; CI = 95% confidence interval; SD = standard deviation. Abbreviation: BMI = body mass index; eGFR = estimated glomerular filtration rate.

Table S22 Association between Right Ventricle function parameters and Left Ventricle function parameters, according to gender

Per SD	Model 1	p-value	Model 2	p-value	Model 3	p-value
Male n=207			**LV End–diastolic Volume**			
RV Ejection fraction	0.68 (-4.05; 5.4)	0.778	0.43 (-4.08; 4.93)	0.852	1.06 (-3.47; 5.58)	0.645
			**LV End–systolic Volume**			
RV Ejection fraction	-6.23 (-8.81; -3.64)	<0.001	-6.29 (-8.84; -3.74)	<0.001	-5.98 (-8.57; -3.4)	<0.001
			**LV Stroke Volume**			
RV Ejection fraction	6.88 (4.08; 9.68)	<0.001	6.69 (4.06; 9.33)	<0.001	7.01 (4.37; 9.65)	<0.001
			**LV Ejection fraction**			
RV Ejection fraction	4.56 (3.54; 5.58)	<0.001	4.58 (3.56; 5.6)	<0.001	4.5 (3.48; 5.53)	<0.001
			**LV Peak ejection rate**			
RV Ejection fraction	16.05 (-5.11; 37.21)	0.136	18.11 (-2.05; 38.26)	0.078	19.91 (-0.22; 40.04)	0.052
			**LV Early diastolic rate**			
RV Ejection fraction	9.04 (-7.9; 25.99)	0.294	11.14 (-4.77; 27.04)	0.169	11.71 (-4.35; 27.76)	0.152
			**LV Late diastolic rate**			
RV Ejection fraction	21.96 (1.31; 42.61)	0.037	21.99 (1.23; 42.75)	0.038	21.76 (1.1; 42.41)	0.039
			**LV Mass**			
RV Ejection fraction	2.6 (-1.33; 6.54)	0.194	2.01 (-1.68; 5.7)	0.284	2.83 (-0.9; 6.55)	0.136

The beta estimate given with a 95% confidence interval represents the estimate size between cardiac right and left ventricle from linear regression model, according to gender. The model 1 = adjusted for age; model 2 = model 1 + smoking, alcohol use, BMI, systolic blood pressure, diastolic blood pressure, diabetes mellitus, total cholesterol and eGFR; model 3 = model 2 + insulin, glucose, antihypertensive medication, lipid lowering medication; CI = 95% confidence interval; SD = standard deviation. Abbreviation: BMI = body mass index; eGFR = estimated glomerular filtration rate.
